# Outcomes of Micro-Dissection TESE in Patients with Non-Mosaic Klinefelter’s Syndrome without Hormonal Treatment

**DOI:** 10.22074/ijfs.2015.4182

**Published:** 2015-02-07

**Authors:** Hakan Ozveri, Furkan Kayabasoglu, Cem Demirel, Ersan Donmez

**Affiliations:** 1 Department of Urology, Acibadem Kozyatagi Hospital, School of Medicine, Acibadem University, Istanbul, Turkey; 2 Istanbul Kosuyolu Gynecology Center, Istanbul, Turkey; 3 Department of Obstetrics and Gynecology, Istanbul Acibadem Hospital, Kadikoy, Istanbul, Turkey; 4 Embryology Division, IVF Unit, Istanbul Acibadem Hospital, Kadikoy, Istanbul, Turkey

**Keywords:** Klinefelter Syndrome, Sperm Retrieval, ICSI, Pregnancy

## Abstract

**Background:**

Klinefelter syndrome (KS) is the most common sex chromosomal disorder in males and historically patients have been labeled as sterile. After the introduction of microdissection testicular sperm extraction (micro-TESE), successful sperm retrievals for intracytoplasmic sperm injection (ICSI) have been reported.

**Materials and Methods:**

A retrospective study was undertaken on ten patients with non-mosaic KS undergoing micro-TESE for ICSI. The testicular volume and FSH and LH levels of each patient were measured. Karyotypes were confirmed by analyzing peripheral lymphocyte metaphases. Physical examination of the external genitalia was performed in all patients to rule out any co-existing anomaly. Micro-TESE was performed in order to investigate the presence of seminiferous tubules which may contain spermatozoa. When testicular spermatozoa were found in micro-TESE, ICSI was performed. Embryos were evaluated for further development. Fertilization was considered to have occurred after the visualization of the two pro-nuclei stage of the oocyte 24 hours after the intracytoplasmic injection of the motile spermatozoa. Pregnancy was confirmed by visualization of an intrauterine gestational sac under ultrasonographic examination.

**Results:**

Testicular biopsy revealed motile spermatozoa in 6 of 9 patients (66.6 %). Fertilization rate per embryo-transfer was 40%. One patient was able to conceive and fathered a healthy boy weights 3410 g at the 39^th^ week of gestation.

**Conclusion:**

Retrieval of testicular spermatozoa by micro-TESE is possible for azoospermic men with KS when assisted reproductive techniques are applied. For patients with KS who want to conceive, assisted reproductive techniques (ART) should be recommended.

## Introduction

First described in 1942 (1), Klinefelter’s syndrome (KS) is a frequent sex chromosome abnormality occurring in approximately 1 in 500-600 phenotypic males (2, 3).

KS is present in 3% of infertile male patients and in up to 11.9% of azoospermic males (4,5). It may present itself in non-mosaic (47,XXY) or mosaic (47,XXY/46,XY) forms, and eighty-five percent of Klinefelter patients have a nonmosaic 47,XXY karyotype (6).

The sexual development of KS patients is normal during the pre-pubertal years.

Many 47,XXY patients appear to enter puberty normally but testosterone concentrations begin to decline in late adolescence and early adulthood (7). A decrease in androgen production prevents secondary sexual characteristics from developing completely, and promotes the development eunuchoidism and gynecomastia. Possible characteristics of the disease can vary, ranging from little or no sign to a lanky, youthful build and facial appearance to even a rounded body type with some degree of gynecomastia. KS patients often have smaller than average testes along with elevated gonadotropin levels.

Testicular histopathologic examination often reveals germ cell atrophy with fibrotic Leydig cell hyperplasia and hyalinized seminiferous tubules (8). Improper functioning of Leydig cells at the onset of puberty causes secretion of high estradiol and low to normal testosterone concentrations, rendering the patient to have elevated levels of luteinising hormone (LH) and follicle stimulating hormone (FSH) (9). A karyotype analysis of peripheral blood, the gold standard for diagnosis, may be necessary in the case of certain physical manifestations such as small testes and penis, sparse facial, body and pubic hair, distribution of adipose tissue resembling female body habitus, diminished libido, gynecomastia, abnormally long limbs, developmental delay, speech and language deficits, learning disabilities, psychosocial difficulties and behavioral issues (10, 11).

Despite the historic belief of males with KS being sterile, advances in assisted reproductive techniques (ART) have resulted in several reports of pregnancies.

In this article we report our testicular sperm retrieval rate and other outcomes of assisted reproductive techniques in non-mosaic KS patients.

## Materials and Methods

A retrospective study was undertaken on ten patients with non-mosaic KS undergoing microdissection testicular sperm extraction (micro- TESE) for intracytoplasmic sperm injection (ICSI). Between January 2006 and May 2009, 13 patients with KS were diagnosed in a private IVF unit. This study approved by Ethical Committee of Acıbadem University. Informed consent was obtained from all patients and ten out of 13 patients agreed to undergo *in vitro* fertilization (IVF) treatment. All males had semen analysis according to World Health Organization criteria to confirm azoospermia. The testicular volume plus FSH, LH and total testosterone levels of each patient were measured. Free testosterone was calculated from the levels of sex hormone-binding globulin (SHBG) and total serum testosterone (T) according to the method described by Vermeulen et al. (12). The blood sample was drawn between 08:00- 10:00 am preoperatively, and 1, 3, 6, 12 and 18 months after micro-TESE. Karyotype confirmation was done by the analysis of peripheral lymphocyte metaphases. Physical examination of the external genitalia was performed in all patients to rule out any co-existing anomaly. Ovulation induction of the female partner was achieved by the combination of a gonadotropin releasing hormone (GnRH) analogue (leuprolide acetate; Lucrin®, Abbott, Aubonne, Switzerland) and human menopausal gonadotropin (hMG) (follitropin beta; Puregon®, Organon, Oberschleissheim, Germany). The gonadotropin dosage was adjusted according to the ovarian response of each individual. Ovulation was induced by 10000 IU of human chorionic gonadotropin (hCG) (Pregnyl®, Organon). Oocytes were collected transvaginally under ultrasound guidance 32-38 hours after hCG administration. Oocyte were harvested synchronously to micro- TESE.

Prior to testicular biopsy, none of the patients with KS had received hCG and androgen replacement therapy. Patients who had received any hormonal or medical treatment pre-TESE were excluded from the study. The patients with KS underwent micro-TESE with all procedures performed by the same surgeon. The procedure of the micro- TESE was performed under general anesthesia. After proper cleaning and draping, a 3 cm equatorial scrotal incision was carried down to the tunica vaginalis. The testis was accessed from this incision and fixed in place by the assisting surgeon. Direct examination of the testicular parenchyma was then carried out at ×20-25 magnification under a microscope equipped with an Olympus modulation contrast system.

The tunica albuginea was incised in the midtestis at an avascular plane, horizontally with respect to the vascular architecture of the testis, to avoid subtunical blood vessel damage. The incision was continued around two thirds of the circumference of the testis to expose all seminiferous tubules. Small veins were cauterized with bipolar cautery but large veins and arteries were carefully preserved. Once the bleeding was controlled, the testis was gently spread open and the seminiferous tubules were exposed. The tubules which were dilated and opaque (considered to contain sperm) were harvested using fine surgical forceps and scissors. Up to 15 pieces of tubules , ranging from 2 to 10 mg, from each testicle were sent to IVF laboratory for immediate examination of presence of spermatozoa. As soon as spermatozoa were identified, no further testicular tissue was harvested. A separate small testicular tissue was taken and sent for histopathologic examination. The tunica was closed with 5-0 Prolene sutures and the rest of the incision was closed with 5-0 rapidly absorbable suture material.

All of the spermatozoa injections were performed without any complications. Fertilization was considered to have occurred after the visualization of the two pro-nuclei stage of the oocyte 24 hours after the intracytoplasmic injection of the motile spermatozoa. In the third day after oocyte pick-up, embryos were transferred transcervically into the uterine cavity under ultrasound guidance. The luteal phase was supplemented with micronized progesterone (micronized progesterone, Progestan®; Kocak, Istanbul, Turkey), 100 mg three times per day, intravaginally. Pregnancy was confirmed by visualization of an intrauterine gestational sac under ultrasonographic examination.

## Results

The mean age of the patients with KS was 33.40 ± 7.65 years (range of 22-49 years). The mean FSH and LH serum levels of patients were 44.11 ± 25.26 mIU/ml and 21.08 ± 5.61 mIU/ ml respectively. The mean basal serum testosterone level was 10.9 ± 2.88 ng/ml. The normal ranges in our laboratory were 1.5-12.4 mIU/ml for FSH, 1, 7-8, 6 mIU/ml for LH, and 10-29 ng/ml for basal serum testosterone. Postoperative FSH, LH and total testosterone levels are shown in table 1. Mean serum T showed an average 27 to 30% decrease from baseline when assessed 1, 3, 6 months after micro-TESE and T returned to 85% of the pre-TESE level after 18 months. FSH and LH levels did not change significantly. All patients had small testicular volumes of 2-5 ml.

Histopathologic analysis of testicular biopsies at the time of sperm retrieval revealed 6 cases with Sertoli cell-only pattern, two with focal hypospermatogenesis and one case with Leydig cells-only. Sperm retrieval rates per attempt at TESE, relative to biopsy histology are given in table 2. Focal hypospermatogenesis was the histologic result in one case which ended up with pregnancy.

Micro-TESE procedure was carried out in 9 (90%) of the 10 patients. In case number 6, micro-TESE was cancelled due to failure in ovulation induction. Testicular biopsy revealed motile spermatozoa in 6 patients (66.6%). Subsequently five out of six couples underwent one ICSI cycle except only case 9 who underwent two ICSI cycles. In case number 7, the two pronuclei stage was not visible 24 hours after the sperm injection. The fertilization rate following ICSI was found to be 40% (26/65) in our study. Cryopreserved spermatozoa was used for only one ICSI cycle (in case number 9) and fertilization was also detected in this cycle. Out of the sixteen embryo transfers only one singleton pregnancy was obtained (in case number 4, Table 3). Overall implantation rate was 6.25% (1/16) and pregnancy rate per embryo transfer was 16.6% (1/6). Preimplantation genetic diagnosis (PGD) was not undertaken for the developing embryos.

In case number 4, pregnancy was achieved and the first ßhCG concentration was 66 IU two weeks after the embryo transfer, followed by a single pregnancy with a visible heartbeat in the sixth gestational week. The results of all prenatal diagnostic investigations were normal, and the woman delivered in the 39^th^ week of her pregnancy by caesarean section, giving birth to a healthy boy weights 3410 g. The karyotype of the fetus was found to be 46-XY by chorion villus biopsy.

**Table 1:** Postoperative serum levels of FSH, LH, and testosterone levels in the follow-up period


	Preop level	Postop 1 month	Postop 3 months	Postop 6 months	Postop 12 months	Postop 18 months

**Testosteron (T)**	10.9 ± 2.89	7.94 ± 2.01	7.7 ± 1.94	7.53 ± 2.05	8.06 ± 2.13	9.41 ± 2.48
**FSH**	44.11 ± 25.26	44.81± 24.93	47.72± 25.47	44.63± 24.44	44.81± 26.13	45.13± 26.51
**LH**	21.08 ± 5.61	23.05± 6.22	23.83± 7.36	24.40± 6.88	23.66± 6.67	23.58± 6.59
**T Change frombaseline %**	-	27.15	29.30	30.90	26.05	13.60


FSH; Follicle stimulating hormone and LH; Luteinising hormone.

**Table 2:** Success of sperm retrieval per attempt at micro-TESE in men with KS analyzed by histopathological pattern of testicular biopsy


Histopathologic pattern	N	Successful retrieval (%)

**Sertoli cell only**	6	4(64)
**Focal hypospermatogenesis**	2	2(100)
**Leydig cell hyperplasia**	1	0(0)


micro-TESE; Microdissection testicular sperm extraction, KS; Klinefelter’s syndrome and N; Number.

**Table 3:** Results of micro-TESE and ICSI


Case	Patient age (Y)	Oocytes	Spermatozoa	MII oocytes	2 pro-nuclei	ET	Pregnancy

**1**	28	17	Negative	13	-	Cancelled	-
**2**	37	Cancelled	Negative	-	-	Cancelled	-
**3**	33	8	Negative	6	-	Cancelled	-
**4**	31	19	Positive	16	4	2	Positive
**5**	34	11	Positive	7	3	3	Negative
**6**	49	negative	Cancelled	-	-	-	-
**7**	38	5	Positive	4	Negative	Cancelled	-
**8**	25	14	Positive	9	3	3	Negative
**9**	22 first ICSI cycle	10	Positive	9	3	3	Negative
	27 second ICSI cycle	27	Freeze	19	12	4	Negative
**10**	37	1	Positive	1	1	1	Negative
**Total**	-	-	-	65	26	16	-


ET; Embryo transfer, micro-TESE; Microdissection testicular sperm extraction and ICSI; Intracytoplasmic sperm injection.

## Discussion

KS is a disorder of the gonad due to an error in meiosis leading to an abnormal karyotype (9). The most prevalent chromosome anomaly, 47,XXY, is diagnosed in 3% of infertile male patients (4), and up to 11% of patients with azoospermia (13). About 15% are mosaic cases, usually with two cell lines 47,XXY and 46,XYand severity increases in parallel with the proportion of the aberrant cell population. The others are considered non-mosaic, upon cytogenetic examination of somatic cell lines (6).

Males with azoospermia and non-mosaic 47,XXY KS were considered sterile in the past. Now, with the advent of testicular sperm extraction and intracytoplasmic sperm injection (ICSI), patients with KS may realize their reproductive potential. The first case of high fertilization rate with ICSI using sperm from a patient with KS was reported in 1995 by Harari et al. (14). Tournaye et al. (15) then reported the first positive TESE in KS in 1996 and two years later, Palermo et al. (16) reported first births after ICSI/TESE in KS. Hinney et al. (17) and Bourne et al. (18) reported the first pregnancy case and delivery from KS patients. After them, several reports of successful ART in these patients have been published during the last decade.

In the literature, some pregnancies have been reported which occurred by the usage of ejaculatory spermatozoa from patients with KS (18, 19). The percentages of successful sperm recovery in these patients were variable. In the reports of Westlander et al. (3) and Madgar et al. (5), retrievals of testicular spermatozoa by TESE were described as 21 and 45%. In these reports, retrieving sperm with micro- TESE has a slightly higher success rate than TESE. Koga et al. (20) and Schiff et al. (21) detected adequate motile spermatozoa in 50% and 72% of micro- TESE procedures in patients. The rate of successful sperm recovery in our study (66.6%) is also in agreement with these results. In the review of the literature, there was no predictive factor of successful testicular sperm retrieval by micro-TESE. Koga et al. (20) detected that the outcome of micro-TESE for non-mosaic KS patients appears to depend on the identification of seminiferous tubules without sclerotic changes in the testicular tissue. In addition Madgar et al. (5) suggested that all patients with KS should receive hCG treatment for at least six months before testicular sperm extraction.

The outcomes of micro-TESE are comparable in patients with KS and non-KS. One of the largest study series on micro-TESE reported the results of 792 procedures, which achieved a sperm retrieval rate of 60% (22). Although we are presenting a small group of patients, our sperm retrieval rate of 66.6% is comparable to the sperm retrieval rates in non-KS patients.

One of the main concerns is the efficacy of spermatozoa retrieved from males with KS in achieving fertilization and subsequent embryo development. In this study, the proportion of fertilization for ICSI was 40%. The fertilization rates in studies by Friedler et al. (6) and Ulug et al. (23) were reported to be 66 and 54.2%. It is therefore apparent that the majority of spermatozoa are able to be effective. We used cryopreserved spermatozoa for only one ICSI cycle and detected fertilization in this cycle. Friedler et al. (6) found no statistically significant difference in the two pronuclear fertilization rates (66 versus 58%) for fresh and cryopreserved sperm.

The pregnancy rate in ICSI of KS patients has been evaluated in several reports. The pregnancy rates per embryo transfer in Ulug et al. (23) and Kahraman et al. (24) studies were detected to be 27.2% and 50% respectively. Also Levron et al. determined the pregnancy rate per embryo transfer to be 50% (25). In our study the pregnancy rate perembryo transfer (16.6%) was lower than that in the previous reports.

The other important concern about KS patients undergoing assisted reproductive techniques is the possible increase in the incidence of chromosomal abnormalities and whether prenatal diagnosis should be performed. Recent studies on chromosome abnormalities in ejaculated spermatozoa from KS patients showed that the incidence of hyperhaploid sex chromosomal sperm cells has increased (24, 26). However, it is established that the percentage of chromosomal abnormalities in peripheral blood cells neither predicts the chromosomal constitution of the testis cells nor the presence or absence of spermatogenesis (3, 9). The ability of 47,XXY germ lines to proceed through meiosis is not certain and several reports have been published on this issue (25, 27). Thus, the risk of producing pregnancies with chromosome abnormalities by using sperm from non-mosaic 47,XXY men is not exactly determined.

Staessen et al. (28) state that the use of PGD to identify the abnormalities in a cohort of morphologically good quality embryos prevents the transfer of those embryos that are destined either not to implant or to abort spontaneously. They also mention that there is an argument against PGD which might have negative effect on the viability of precious embryos which were generated after ICSI and sometimes with extremely rare sperm. But as they stated, no convincing evidence has been produced so far that would demonstrate a possible dramatic effect of biopsy on the implantation potency of the embryo. Greco et al. (29) concludes in their very recent study that PGD on embryos conceived with sperm from men with non-mosaic KS is not strongly indicative since they reported sixteen babies with a normal karyotype. On the other hand, they recommend that until conclusive information is available, such couples should be offered extensive genetic counseling.

One other important aspect of micro-TESE in patients with KS is the preservation of testicular tissue mass and avoiding deterioration of the androgenic status of the patient. In our study, in patients who underwent micro-TESE, mean serum T showed an average of 20-25% decrease from baseline when assessed 3, 6, 12 and 18 months after micro-TESE. T returned to 85% of the pre-TESE level after 18 months. Although this return may be attributed to the preservative nature of micro- TESE procedure, such possible declines should always be kept in mind in performing micro-TESE in patients with very small volume of testicles in order to prevent further deterioriation of gonadal status of these patients.

## Conclusion

Patients with KS can be offered micro-TESE-ICSI treatment. Even the patients who were labeled sterile historically, such as non-mosaic 47,XXY KS, benefit from micro-TESE-ICSI procedure and men with KS can father healthy children. Although we presented here a very small number of patients who had many bad prognostic factors such as limited testicular volume, high FSH and/or very low testosterone levels, sperm retrieval was possible in 66.6% in micro-TESE attempts. The use of micro- TESE for men with non-mosaic KS had a high sperm retrieval rate, comparable to that seen for other men with nonobstructive azoospermia. On the other hand, hypogonadism which may become worse in patients with KS after even microdissection should always be taken into consideration in the follow-up of these patients.
